# Cultivating Health in Martial Arts and Combat Sports Pedagogies: A Theoretical Framework on *the Care of the Self*

**DOI:** 10.3389/fsoc.2021.601058

**Published:** 2021-03-04

**Authors:** Lorenzo Pedrini, George Jennings

**Affiliations:** ^1^ Department of Sociology and Social Research, University of Milan-Bicocca, Milan, Italy; ^2^ Cardiff School of Sport and Health Sciences, Cardiff Metropolitan University, Cardiff, United Kingdom

**Keywords:** care of the self, cultivation, health, martial arts and combat sports (MACS), multimodalily, pedagogies, praxiography, wellbeing

## Abstract

“Martial arts and combat sports” (MACS) are a myriad of systems of embodied movements and underlying philosophy and pedagogies. Due to the intrinsic complexity of MACS, they have the potential to both reshape practitioners’ selves and improve their wellbeing, as well as to hamper the pursuit of sustainable, healthy lifestyles. This article provides an interdisciplinary theoretical framework to critically approach both the “light” and the “dark” sides of martial pedagogies. The model we propose develops the Foucauldian notion of “*the care of the self*,” which has been considerably overlooked in martial arts scholarship. Furthermore, by viewing health as a goal for *cultivation*, this proposal places the situated practices linked to materiality and discourses at the centre of the theoretical and empirical analyses. The article thus takes into account the internal diversity and cross-institutional variance of martial pedagogies by allowing scholars to explore four forms of cultivation (*self, shared, social, ecological*) prompted on a day-to-day basis. To conclude, we discuss the main methodological implications for multimodal research arising from the framework in order to foster future inquiries.

## Introduction

Without doubt, with the advent and spread of the COVID-19 virus, the topic of health has become dramatically important in global society. National governments are trying to face the crisis, while the World Health Organization (WHO) is striving to limit the diffusion of the fatal virus. Universities, as well as national and private research centres have developed forms of anti-virus and have begun to contrast ― or understand, at least ― the ongoing social changes resulting from the pandemic. Our daily routines, relationships and lifestyles have been reshaped in unpredictable ways.

As “martial arts and combat sports” (MACS) (Channon and Jennings, 2014) aficionados based in two Western countries, we are aware about the impact of COVID-19 on martial communities, as we have also experienced how some organisations have reacted to face the health crisis^1^.

For instance, certain Taijiquan (Tai Chi Chuan) and historical European martial arts (HEMA) schools in UK have implemented online classes, film nights and fitness regimes (*see* Jennings, 2020). This illustrates the specific responses use to maintain physical fitness and muscle mass for distinct combat sports advocated in Spain, as in wrestling and Judo (Herrera-Valenzuela et al., 2020). During the lockdown stage (March-May 2020), most of the Italian people’s grass-roots boxing gyms (*palestre popolari)* – themselves created in abandoned squatted buildings – were turned into warehouses to stock food and medicines. Many *boxe popolare* practitioners joined the welfare programmes of the local municipalities for supporting citizens in need ― e.g., isolated elderly people without relatives, unemployed adults and disabled people. Last but not least, the Italian feminist boxing team named *le sberle* (“the slappers”) run the campaign ‘No One’s Alone’ in order to collect money for sex workers ― unable to work due to mobility restrictions ― and to sensitize public opinion about these sex workers’ (vulnerable) condition via social media.

As these examples suggest, the relationship which intertwines MACS cultures with the issues of health and wellbeing is multifaceted, and, we do not want to deny, controversial. Nonetheless, given the current debate within the interdisciplinary field of “martial arts studies” (see, in particular, Bowman, 2018), the question “how might martial arts and combat sports be good or bad for health?” has yet to be framed and tackled in any explicit way. This is starting to change, however, with new empirical studies on martial arts, health and society as seen in this special issue of *Frontiers in Sociology*. A notable contribution to empirical data is the recent monograph from Fuller and Lloyd (2019) sharing adult practitioners’ viewpoints (through a large-scale survey and in-depth interviews) on the subjective aspects of wellbeing gained from the sustained practice of a variety of martial arts (but particularly Taijiquan and Karate) in several countries. For their broad range of participants, Fuller and Lloyd (2019) identified numerous benefits of martial arts training such as dealing with back pain and postural problems. However, this research remains exploratory in nature and tends to avoid any detailed theorising around the questions of pedagogy, embodiment and transformation. This is quite understandable, as health is an extremely complex theme to be conceptualised and researched, with various competing health philosophies and pedagogies regarding the huge spectrum of MACS (Jennings, 2014). On the one hand, martial arts and combat sport exercises may “help people become fitter, defend themselves, feel more relaxed and develop specific personal and cultural values” (Jennings, 2019, p. 137). On the other hand, to continue following Jennings’ account of the “light” and “dark” sides of martial arts activities (Ibid, p. 138), “many practices are outdated, risky and misguided [...] with drills and exercises that are not compatible with a sustainable, healthy lifestyle.”

The aim of this article is to provide a conceptual framework for critically approaching the *(un)healthy pedagogies* embedded in MACS practice. The critical pedagogy we call for consists of a balanced viewpoint about “both the seemingly healthy practices and the potentially unhealthy, even dangerous, practices in a given physical culture by considering their internal diversity and cross-institutional variance” (Jennings, 2019, p. 138)^2^.

In the wake of the spirit of the burgeoning field of martial arts studies, which embraces anthropological, cultural, historical, philosophical and sociological inquiries (Bowman, 2015), as well as other visions for research into the world’s armed and unarmed fighting systems in specific regions (Cynarski, 2012; Ryan, 2020), our ambition is to build a definition of health beyond a purely physiological or biomedical paradigm. Our viewpoint on health therefore crosses the disciplinary boundaries and unifies different perspectives within the paradigm of embodiment by focusing on practice, that is, “what is taught (the movements) and how (the methods) – in essence, the practices: the nexus of not only doings, but also the sayings” (Jennings, 2019, p. 318).

Making use of ideas around self-cultivation and care of the self, we combine perspectives inspired by the Japanese philosopher Yuasa with the celebrated French thinker Michel Foucault. This combination is both novel and perhaps necessary given the global dimension of MACS, the calls to break down disciplinary boundaries in martial arts studies (Bowman, 2015) and the strange absence of Foucault’s ideas in this emergent field – something noticeable given the special place of his works in sport studies and physical culture studies.

The paper is structured in three sections: the first and main part of the article reviews the main research findings by elucidating the overarching perspective of embodiment in order to frame the (un)healthy pedagogies of martial arts and combat sports. Here, we discuss the Foucauldian notion of ‘the care of the self’ (Foucault, 2005) and introduce the idea of cultivation. The second section outlines four interconnected dimensions of cultivation (*self, shared, social* and *ecological*), in which health might be conceptualised, developed and investigated. The third section offers some methodological insights for future (interdisciplinary) research. The methodology relies on the approach of “praxiography” proposed by Bueger (2014), whose main aim is the actual analysis of specific practices through data collection and interpretation strategies. In conclusion, we introduce a few questions implicated in our article for other researchers and practitioners to consider.

## Towards a Theoretical Framework Based on Embodiment

Widely speaking, pedagogy can be considered as the ensemble, more or less codified and explicit, of the principles, the precepts and the methods to transmit skills, values and norms, so as to reshape human beings and how they perceive and act in the world.

Inquiries into MACS from the discipline of sociology have devoted significant attention to this topic over the last few decades, paying particular attention to specific dexterities and cultural values in cross-cultural contexts (Channon and Jennings, 2014; Delamont et al., 2017). As international texts such as that of Sánchez García and Spencer (2013) have shown, these ethnographic investigations pertain mainly to gender, culture, identity and violence through the apprenticeship into a particular martial arts subculture. These have drawn extensively on the theories of Bourdieu and Elias, in particular in terms of the notion of habitus, as well as on the concept of “techniques of the body” coined by Mauss (1973). These concepts are milestones in approaching embodied experience in MACS around the world (for a review, see Channon and Jennings, 2014).

More exactly, embodied experience ─ or, embodiment ─ refers to the living, moving, active body with agency for self-reflection, improvement and healing (martial arts as forms of medicine and healing in themselves), but, for our purposes, not one involving external surgery for aesthetics (for a sharp contrast, see Orbach’s (2008) account of how bodily ideals in global modernity have turned many people to psychotherapy and cosmetic surgery). Embodiment is also the key to what Eichberg (1998) terms significantly as body cultures: the elements of broader culture centred around movement. In this view, body cultures are always pluralistic, offering multiple possibilities in different spaces and across time. They can be for performance, health and also cultural expression (and quite often a shifting mix of the three positions). For instance, over the centuries, fencing has shifted from a battlefield activity and one for duels to the death to a modern Olympic sport. Yet there has been resistance to the sportisation process and a renaissance of interest in historical fencing and lost styles known as the historical European martial arts (HEMA), which are being researched, re-enacted and revived through the creation of new materials, organisations and events (Jaquet et al., 2020). The techniques and objects are recreated in modern times, which enables us to consider the potential benefits and risks associated with wielding a powerful weapon such as a longsword or a poleaxe.

In addition, two very different inquiries on the pedagogies of two very diverse practices and milieu ―*Kalarippayattu* in South India and *Juego de Garrote in* Venezuela ― have demonstrated that martial arts can also be adopted for healing purposes to improve practitioners’ self-confidence and feeling of power, which all stems from the preparation, curation and training of the body (Zarrilli, 2000; Ryan, 2016). Accordingly, Jennings (2014) demonstrates how practitioner-instructor interactions in Wing Chun Kung Fu and Taijiquan associations transmit precise health philosophies from Daoist, New Age spiritualities and Western medicine. This is assisted by the multicultural nature of contemporary Britain in an information age, where alternative views on the body and health are readily available. Yet the influence of health philosophies and medical paradigms on martial arts training is not new, as seen in the *Yojokun* (Kaibara, 2008); a 18th-century text written by a Japanese Samurai influenced by the classic texts of Chinese medicine at a time of increasing peace in that then feudal society. As an early self-help or self-care book, the *Yojuken* offers a glimpse into how themes of longevity, and specific techniques to maintain particular areas of the body (as in Bates’ (2019) recent work in the British secular context) are vital for a holistic model of health.

However, despite the relevance of these studies on culturally-distinct MACS, several questions remain. How do specific conceptions of health emerge in a broader set of martial arts and combat sports? How do different aspects of health complement each other within a given bodily art/sport discipline? How are they transmitted and acquired in different socio-cultural environments, traditions and across federations?

Recently, seeing theory as central to any discipline, Bowman (2017) has called for a central place of theorising in martial arts studies, and this further conceptualisation could also have a role in studies pertaining to health. In the following two sections, we hence move towards a more detailed (theoretical) definition of health that overcome a purely (bio)medical paradigm. The first step consists in discussing the concept of “the care of the self” (*epimeleia heautou*) (Foucault, 2005), which surprisingly, given the popularity of Foucault’s perspectives and concepts in sport studies, as well as the more recently established physical culture studies (Silk et al., 2019), represents a novelty in the field of MACS scholarship. For our purposes, the notion of “the care of the self” seems particularly pertinent since it implies embodiment. Besides, the notion allows to explore the physical, symbolic and relational dimensions of health and wellbeing, furnishing the basis to further empirical (interdisciplinary) inquiries.

Last but not least, one of the focal points of the “care of the self” is pedagogy and how it is articulated in practice in situated, organised environments, that is, the daily teaching and learning process ─ i.e., education in all its guises. This might include regular classes, informal training between classmates, training at home or in a park, private lessons between a teacher and their student, seminars with lineage holders and workshops with specialists and competitions between schools: all of which are used to varying degrees in different MACS systems.

### Care of the Self

The well-known French philosopher and social historian Michel Foucault introduces the notion of the care of the self in the final stage of his works ― also defined as the “ethical turn” ― in relation to this idea of subjectivity: how individuals shape and transform themselves in order to reach a given state of grace, happiness and wellbeing (Foucault, 2000; Smith, 2015).

The concept, and the late Foucault’s investigations, are extremely significant because they break with his previous and most popular arguments, according to which the conception of health ― with particular reference to modern West of the last few centuries ― would relate to the medical gaze and social institutions, whose main scope is to produce docile bodies for the maintenance of social order (Randall and Munro, 2010). Primarily through the workings of science and the related development of institutions such as mental asylums, jails, schools and hospitals, subjects have been divided into opposite categories, e.g. “the mad and the sane, the sick and the healthy” (Foucault, 1983, p. 208).

Accordingly, drawing on Dunning and Waddington’s (2003) theorisation, two different approaches to sporting culture has emerged: the “Stoical/Puritanical” and the “Epicurean/Dionysian,” with the former emphasising the hygienic/disciplining aspect of body cultures, the latter more concerned in boosting hedonistic experience. In particular, the Puritanical approach “received clear expression in the 19th century, with the formation […] of the *mens sana in corpore sano* ethos, that was bolstered in the wider society by the emergence of the so-called “rational recreation” movement and, what might be called the “sport/health” ideology,” which has remained hegemonic in Western cultures for two centuries. In contemporary postmodern times, this ideology still permeates a wide range of body cultures, even informing social policies.

The ethical turn of Michel Foucault, evolved since the mid-Seventies, responds to the opposing transformations occurring in the global political and social scenario: on the one hand, the consolidation of neoliberal societies and new forms of bio-power; on the other hand, the rising of ‘new social movements’ (Melucci, 1996), which criticize and resist the governmentality processes in several realms of daily life.

Over the last three decades, the collapse of the Welfare States and the achievements in the field of social and civic rights have been entailing to the fragmentation and lability of the modern discursive regimes, habits and customs (Alston, 2017) ― in the field of health, too. In late modernity, borrowing Giddens (1991) term for this period, individuals’ lives are less pre-determined, as they live in a condition of ontological uncertainty. Environment, culture and society change rapidly and affect human-beings in several ways. Because of the social order dynamism, the selves are always at stake. Everyone becomes the main responsible for her/his own destiny, for her/his own wellbeing, successes and failures (Mellor and Shilling, 1993) – and even self-reinvention at a time where the increasingly individualised self is expected to readily adapt to societal expectations, as the fitness culture demonstrates (Sassatelli, 2010; Elliot, 2012). Martial arts could play a role in such a society, with the growing number of self-help books (e.g., Jones, 2004; Thompson, 2010) encouraging individuals to take full responsibility for their destinies, including the care of their own bodies as seen through Chinese, eclectic or Western medical perspectives.

Being sensitive to those trends of individualism and neoliberalism, Foucault framed the “care of the self” (from the Ancient Greek concept of *epimeleia heautou*) as an ethical project based on experience, the relation with one-self and to others; and it is not run by any law or stable institutions (Smith, 2015)^3^. Care of the self, then, might include personal hygiene, massage, healthy sleeping patterns, regular hydration, walking in the fresh air and taking a holiday from work. In martial arts, this could include the various forms of massage and bone setting that accompany specific traditional medicine and healing systems (*see* Zarrilli, 2000; Cynarski, 2012), or the regulation of temperature during training and warm-ups/cool-downs, boxing workouts, standing postures in traditional Chinese martial arts and even using thermoception to maximise the execution of a mixed martial arts technique (Allen Collinson and Owton, 2015; Allen Collinson et al., 2016).

In this sense, care of the self postulates a sort of physical immanence and points its difference with transcend wellbeing and self-realization ― for instance, the “ultimate” or idealized human beings prompted by revealed religions and/or the political ideologies. According to this view, the self is a balance between the body and the mind: “self” means postures, feelings, instincts, guts and thoughts. To emphasise the creative nature of the care, Foucault uses the term “aesthetics of existence,” “whose form cannot be given in advance” (Smith, 2015, p. 135), as the subject is a never-ending project that progresses over the life-course.

Like many of Foucault’s original ideas and notions, “care of the self” is not defined precisely. Foucault prefers a set of evocative expressions to refer to it. In the 1981–1982 Lectures at the Collège de France (The *Hermeneutics of the Subject*) (Foucault, 2005, pp. 8–9), care of the self is termed as:

“a sort of thorn which must be stuck in men's [sic] flesh, driven into their existence, and which is a principle of restlessness and movement, of continuous concern throughout life […] Generally speaking the principle that one must take care of oneself became the principle of all rational conduct in all forms of active life that would truly conform to the principle of moral rationality.”

The notion implies three interlaced aspects that are extremely significant for our theoretical enterprise; more exactly: 1) the care of the self as general (critical) attitude; 2) the care of the self as spiritual awareness; 3) the care of self as embodied activities.

#### Care of the Self as General (Critical) Attitude

First of all, the care of the self means “a certain way of considering things, of behaving in the world, undertaking actions, and having relations with other people. The *epimeleia heautou* is an attitude towards the self, others, and the world” (Foucault, 2005, p. 10). Such an attitude ― in other words, a philosophy ― provides the general framework to conduct an active life that refuses the status quo. The care, from this standpoint, is a sort of twofold “combat” (Berni, 1995). It represents a rejection of the given social reality. In addition, as a principle of living and acting, the care is a sort of self-critique; a way to disapprove ourselves, who and how we are as human beings. As Foucault himself states, through the care of the self “[w]e have to promote new forms of subjectivity while refusing the type of individuality that has been imposed on us” (Foucault, 2000, p. 208). This general framework represents the cornerstone for an active philosophy necessary “to attain a certain state of happiness, purity, wisdom, perfection, or immortality” (Ibid, p. 225)^4^.

In order to achieve this status in the fields of sport and physical culture, certain conditions are required: “An individual must problematize the limitation of his/her current identity; s/he must think critically about being an athlete, a physically (in)active woman, a sports fan, a coach or a health professional advocating physical activity” (Markula and Pringle, 2006, p. 153). This framework or attitude might be seen in the martial arts influenced by other ancient philosophes, such as the path of Daoism taken by many “internal” Chinese martial arts schools such as Taijiquan and Baguazhang, alongside Japanese Aikido, which often has spiritual connotations with Shintoism.

#### Care of the Self as Spiritual Awareness

Second, and consequently, “the care of the self implies a certain way of attending to what we think and what takes place in our thought” (Foucault, 2005, p. 11). This can be defined as the spiritual dimension of the care of the self, that is, a specific and aware relationship that the subjects establish with what they consider as the “truth” or to be meaningful. The care of the self, in these terms, means how we deal with the surrounding social world; how we assess and interiorize representations and norms of conduct; how individuals develop their interior consciousness.

To use a metaphor: care of the self is a glance, a sight towards interiority. The ancient Greek philosophers consider the care of the self as “medicine for the soul” (Iftode, 2013, p. 80), consisting in an ongoing check into personal feelings and thoughts for their modification: that is why “self-care, in Foucault, means ethical and social practices whose goal is to favour the self-fashioning of individuals and/or a spiritual conversion of a sort” (Iftode, 2013, p. 76; White, 2014).

It is important to specify that self-knowledge is the basis for conversion, embracing the subjects as a whole: their thoughts, feelings, bodily postures, and the kind of relationships they are able to establish with others. They all are part of the ongoing embodied project of the care of the self that might have been seen in Ancient Greco-Roman wrestling that is quite removed from the modern approach to wrestling seen in the Olympic Games today. Other forms of wrestling, as in classical anthropological studies in Northern India, seem to have continued this monitoring of thoughts and feelings and control of sexual desire and behaviour (see Alter, 1992).

#### Care of Self as Embodied Activities

Third, then, the care of the self significantly “involves a series of practices, most of which are exercises that will have a very long destiny in the history […] These are, for example, techniques of meditation, of memorization of the past, of examination of conscience, of checking representations which appear in the mind and so on.” (Foucault, 2005, p. 11). In order to be realized, the care of the self requires discipline, i.e., the *askesis*. “Through the rigors of *askesis*, the self can be rendered an object of analysis and hence a critical position external to the self can be achieved” (Alston, 2017, p. 95).

This aspect of the care of the self echoes another one of Foucault's (2000), (p. 87) concepts: the “technologies of the self,” which Foucault defined as “the procedures, which no doubt exist in every civilisation, suggested or prescribed to individuals in order to determine their identity, maintain it, or transform it in terms of a certain number of ends, through relations of self-mastery.”

This quotation introduces pedagogy, the relation with meaningful others and (repetitive) training. Indeed, “care of the self is about […] “living coherently”; it also means a regulated form of existence, the harmony between the words and the deeds, instilled through a series of “techniques” (Iftode, 2013, p. 78), which requires the support of a community of practice and deploys in regulated activities. In other terms, the care of the self implies a series of (disciplined) embodied practices, which are mandatory to concretely realise both sociocultural and spiritual transformation^5^. Many contemporary martial arts systems such as Aikido are even designed to benefit the individual and society through a spiritual philosophy through their techniques and practices. We explore such techniques and practices in the next two sections.

## The (Un)Healthy Pedagogies of MACS and the Idea of Cultivation

The discussion of the care of the self – in terms of a general philosophy of changes, interior awareness and ruled activities – allows us to develop an intercultural idea of human flourishment and cultivation. The theoretical idea of cultivation can be empirically explored in terms of how the “practical pedagogic logic” (Brown and Jennings, 2013, p. 41) of MACS foster perspectives of wellbeing at different levels. Precisely, we propose four forms of cultivation that future research projects could investigate critically, considering these levels separately as well as interlaced. We define these forms as *self-cultivation, shared cultivation, social cultivation* and *ecological cultivation*.

### Self-Cultivation

Inspired by the Eastern mind-body theorist Yuasa (1987) and Yuasa (1993), we define the process of self-cultivation as the long-term, even lifelong, development of human beings through an intensification of mind-body relationships and subsequent development of character and instilling of values. This form of cultivation resembles the care of the self as spiritual awareness. In this vein, previous exploratory enquires have demonstrated how, in particular the commitment to Eastern martial arts and to Latin American body cultures – as in the case of Afro-Brazilian Capoeira – help individuals to create a deeper and more aware relationship with themselves, providing them the opportunity to master bodily skills, develop personal interests and define their social identity in accordance with the nature of the practice carried out (*see*: Spencer, 2011; Channon, 2012; Brown and Jennings, 2013; Delamont and Stephens, 2019).

Indeed, from a critical angle, Spencer (2012) pays attention to the experience of pain among MMA practitioners. He demonstrates the clearly *ambivalent*, as well as unhealthy, relationship established by the fighters in the mid- and long-term between bodily injuries and the normative masculinity connected to the practice. While experience of pain and injuries are considered mandatory pedagogical experience in order to embody a masculine self; “addiction” (Dunning and Waddington, 2003) to exercise and debilitating bodily injury fails to materialise masculine ideals associated with participation in combat sport (for similar considerations on Capoeira, see Stephens et al. in this Special Issue).

### Shared Cultivation

In relation to self-cultivation, with the term shared cultivation we place emphasis on the forms of collective development within formal and informal pedagogies of many systems such as the traditionalist Chinese martial arts (Jennings, 2010). This is akin to the relational element of care of the self. As we have seen, MACS pedagogies indeed do not only reshape individuals. They produce shared cultivation when practitioners are bounded together, create schools that transmit certain abilities and cultural values across generations and practitioners’ lineages. This is due to the techniques of the body (Mauss, 1973) that characterise the arts. Techniques of the body, as the ways in which the bodily movements are adopted and learned in organised social environments, are forms of “primitive classification” (Durkheim and Mauss, 2010). In other words, these classifications serve to bind people together and differentiate social groups.

This can be noticed considering practices ruled very differently, across time and space; for example, Kung Fu practitioners often understood their increasingly intercontinental and intergenerational associations as “families” forming “family trees” or lineages operating across centuries (Partiková and Jennings, 2018). In the case of Italian *boxe popolare* – a contemporary, self-managed boxing style run by leftist grassroots groups outside the jurisdiction of the Italian boxing federation – one of the main scopes of the daily routine is to engage new practitioners within the coaching structure (Pedrini, 2020). The pedagogy of shared cultivation, in other terms, offers a sense of belonging, care towards teammates or classmates and solidarity within diverse communities.

Abramson and Modzelewski (2011) point out that theories of subculture normally explain how people from working-class or marginalised groups join and create subcultures. This would work well with the example of the leftist boxe popolare in Italy, but not for all MACS settings. Abramson and Modzelewski’s (2011) dual ethnographic study shows how MMA gyms act as voluntary subcultures for conventionally affluent and successful middle-class members who do not often come from marginalised or deviant groups in wider U.S. society. Their collaborative ethnography shows the positive side of this voluntary community of men and women of different sizes and shapes who uphold American ideals. This can transfer to ideas around cultivation, as the fighters’ shared values such as commitment and self-improvement enabled interactive levels of cultivation.

A critical attention to shared cultivation, however, has to carefully consider how certain ruled activities can, without being necessarily intended, exclude potential practitioners in terms of age, social class, ethnicity, gender, bodily condition, religious or political beliefs (*see*: Nardini 2016; Delamont et al., 2017; Nardini and Scandurra [forthcoming]). Indeed, it is through the acquisition, transmission and usages of the body that individuals take place in the social world and define who they are – e.g., as youngsters, males, females, and so on and so forth (Bourdieu, 1985). Shared cultivation is hence a way to differentiate the populations and their systems of believes in relation to every aspect of the daily life. We next turn to the system of beliefs, ideologies and politics that govern many MACS cultures, their missions and visions.

### Social Cultivation

We define social cultivation, in preliminary terms, as the processes of human transformation and transcendence occurring in local communities, in specific (sub)cultures, and even in broader society. Learning arts involve the transmission of cultural values and symbolic meanings, which differ across socially organised contexts and practices. Furthermore, pedagogies is the main medium for the transformation of individuals, opening up the opportunity to spread certain perspectives of wellbeing while shaping individuals in a way instead of another. As historical research projects demonstrate, traditional Eastern martial arts as in the Southern Chinese forms of Kung Fu were codified for this scope purposefully (*see* Judkins and Nielson, 2015).

Given the variety of contemporary MACS trans-cultural practices, their pedagogy can be adopted for social justice projects – for example, improving wellbeing of vulnerable populations^6^. Social cultivation underlies a series of political implications in today’s society, which include health issues such as weight or anger management. Then, some MACS pedagogies are instead designed to include people in the realm of civic activism involving them in projects and campaigns for progressive societal change (Pedrini, 2020); while several practices and daily (ruled) interactions, even if they are not designed for explicit political purposes, possess pedagogical significance: for example, in the UK, combat sport classes are sites where cultural subversion to gender order can be experienced (Channon, 2013; Channon, 2014; Channon and Phipps, 2017); meanwhile, in the broader English-speaking world, some male-dominated tribes such as mixed martial art (MMA) communities are increasingly open to include homosexuals (Matthews and Channon, 2015).

Of course, MACS pedagogies remain controversial, since they can rehearse hegemonic discourses and established forms of discrimination. Martial arts can still be used to support political and military ventures whose main aim is to realize supremacist projects, as the rise of the fascist fight clubs in several Western countries and Post-Soviet countries demonstrate (Pedrini et al., 2019). However, despite the political makeup of a country, people are increasingly aware of the environmental consequences of human action. The following notion of ecological cultivation is an extension of cultivation beyond the human and societal into the environmental realm in which they coexist.

### Ecological Cultivation

Ecological cultivation is another layer we include in the analytical proposal. We consider this level of cultivation as a sort of environmental awareness, sensitivity and action. The analysis of the art of Taijiquan in a UK context, for example, illustrates how martial practice can “sow the seeds of environmental awareness” (Brown et al., 2014, p. 380) – the relation with nature is indeed one of the dimension of the care of the self and the pursuit of happiness discussed by Foucault (2011). This form of cultivation draws on Kasper's (2009), (p. 318) definition of ecological habitus, which “refers to the embodiment of a durable yet changeable system of ecologically relevant dispositions, practices, perceptions, and material conditions—perceptible as a lifestyle—that is shaped by and helps shape socioecological contexts.” Also, Eichberg (1998) has emphasized the green attitude of certain vernacular forms of wresting in Scandinavian countries, since these body cultures are strongly related to the cultivation of ethnic identity; wellbeing perspectives prompted by these bodily arts also consists in caring about the natural environment, considered as a fundamental aspect of the local community growth.

This point to the importance of considering the MACS setting, where they are carried out and how they develop implicit pedagogies towards the surrounding environment and the kind of relationship and sensibility the practitioners create with the urban as well as rural landscapes. For example, the Japanese art of Ninjitsu is experiencing something of a renaissance as it is reimagined for different purposes. No longer is Ninjitsu used to train assassins collaborating with the warrior nobility; instead, the modern Ninja can learn their craft at university, with a new master’s course in Ninja Studies. The BBC has followed its first graduate, Genichi, a mature Japanese man who praises the feudal way of living from the earth as a farmer first, and martial artist second, which he believes leads to a sustainable lifestyle within urbanized societies experiencing a pandemic^7^. Other martial arts have specific ecologies and cosmologies embedded within their philosophies and pedagogies, such as the ritualistic forms of Mexican Xilam saluting to the earth (before acknowledging one’s surroundings and neighbours) or the movements of Taijiquan inspired by animals, elements and the cosmos (with poetic and metaphorical terms like “white crane spreads wings,” “cloud hands” and “repulse the monkey”). But how might we study such language and daily talk in MACS pedagogies? This is explored in the next section, which leads to a discussion on praxiography and multimodal research.

## Implications for (Interdisciplinary) Research

In this last section, we provide a few methodological guidelines in order to approach the (multi-layered) topic of health according to the framework proposed. Considering MACS as a set of specific practices, we call for interdisciplinary inquiries and a mixed methodology (Jennings, 2019) as demonstrated by Fuller and Lloyd’s (2019) broad yet exploratory study. The unifying broad perspective we embrace is one of praxiography. To put it simply, it means the analysis of social practices. Praxiography understands “practices as meaningful, regulated bodily movements, which depend on a related implicit incorporated knowledge. Since the majority of practices deal with artefacts (e.g. writing requires a pen and paper), practices are often routinized patterns of behaviour using artefacts. Often a certain way of doing is inscribed into artefacts and they hence can equally be considered as carriers of practices” (Bueger, 2014, p. 387). The main objective of praxiography is to develop a theoretically-driven body of knowledge. According to Littig (Littig, 2013, p. 458), “praxiographic research places the interwoven, supra-individual social practices linked to materiality in the centre of empirical and theoretical analysis.”

For this purpose, we view MACS practice drawing on the definition of (Reckwitz, 2002, pp. 249–250):

“A “practice” (*Praktik*) is a routinized type of behaviour which consists of several elements, interconnected to one other: forms of bodily activities, forms of mental activities, “things” and their use, a background know-ledge in the form of understanding, know-how, states of emotion and motivational knowledge.”

From this view, social worlds and subjects “co-emerge” (Sánchez García and Spencer, 2013, p. 190) in relation to specific martial practices. Specific perspectives and experience of health, intended as forms of cultivation, hence depend on the specific embodied pedagogic logics characterizing each martial art and combat sport in given sociocultural context and institution.

### A Multimodal Approach

The perspective of praxiography to explore the complex relationships between care of the self and cultivation advocate a creative usage of different strategies for empirical research. Even though we do not seek to limit the potential eclectic methodological mix for interdisciplinary research, at this point, we seek to clarify some path that could be followed for investigations in order to stimulate a creative mix of data collection and analysis. Importantly, we emphasise the opportunity to approach practice from a synchronic or a diachronic standpoint through a multimodal approach.

A special issue of *Qualitative Research* (Dicks, Flewitt, Lancaster and Pahl, 2011) has imagined the possibilities of uniting the well-established ethnography (particularly popular in MACS research) with the multimodal research approach, which originally developed in semiotics and linguistics. This multimodal research draws on a range of data collection techniques and data sources in order to understand human and social meaning as opposed to social order. This might be enacted through the exploration of symbols and visual concepts in martial arts theory as seen in badges and crests of different organisations. An example of this is in the world-famous Yin-Yang symbol used in many uniforms and logos, which can be used to explain Daoist health philosophies in the face-to-face teaching of Chinese martial arts in classes and seminars – quite often using whiteboards or physical demonstrations to aid students’ understandings of the oral messages. The meanings behind these symbols and concepts could thus be explored through observation, interviews and semiotic analysis in many other fighting systems. Channon et al.’s (2020) study recounts such a multimodal approach in ethnographic research through their multi-sited fieldwork in conjunction with interviews with a range of medical professionals, coaches and combat sport athletes, which helped them compare official messages around health with the real-life practices in preparation for, within and as a consequence of, competition. In a shocking revelation, this research team revealed a common, even guaranteed, expenditure of ring girls over that on reputed medical professionals.

This above examples offer a critical perspective on pedagogy Markula and Pringle (2006). Given our attention to the discourses in this article, we wholeheartedly advocate studies on how martial arts instructors (be they coaches, *sifus*, *senseis*, *gurus* or *gurukkal*) use specific forms of language within asymmetrical power relations. We wish for instructors to develop a more “ethical guidance” rather than a pedagogy of mastery and self-control, creating actual strategies to negotiate their values, norms, and believes in order to improve self-awareness and critical attitudes of the practitioners so as to improve their abilities to cultivate care (in terms of self-cultivation, shared cultivation, social cultivation, as well as ecological cultivation). Yet at the same time, we are aware of the continued need to study unhealthy and damaging practices such as forced fights, the falsification of medical records and poor hygienic practice during pandemics such as COVID-19.

Depending on the culture of the nation-state and the subculture of the art in question, these coach-athlete relationships and group hierarchies will vary tremendously, although like MMA as other forms of physical culture such as surfing, these MACS are very often based around hierarchies and archetypes of emotive masculinity expressed in mundane daily talk (Green and Evers, 2020).

The links between power, space and emotions could be applied in studies on health in terms of the emotional side of pain, injury, recovery and retirement – especially in more physically demanding combat sports such as MMA or the emotionally challenging approaches taken in Israeli Krav Maga and Russian Systema, which work with ideas around survival psychology and breath control. Talk and emotions can therefore be a key aspect of raw data, which might include the use of humour and changing room banter, text messages, everyday corridor conversations and coaches barking orders from the corner of a ring.

Moreover, adopting a different theoretical perspective, Winchester and Green (2019) explain how the past, present and future social actions are connected through accounts of motivations. They make use of a range of theoretical traditions from hermeneutics to pragmatism to explain how and when people’s accounts in interviews and observations relate to the social context they operate in. The authors argue that the subjective motivations are both “in” the practitioner as well as “outside” them. This adds to the limited research on *talk* in the martial arts, an approach which could enable scholars to understand the motivations behind healthy and unhealthy practices that are not purely individual or institutional, but are certainly driven by the biographies of the participants. In general, approaching practice synchronically allows researchers to grasp the articulation of (un)healthy pedagogy in the making, by adopting the huge spectrum of data collection and analysis adopted in social sciences. The pedagogy of single case-studies can be explored through participant observation adopting several forms of involvement into the field in order to analyse daily interactions (*see* Wacquant, 2013). Martial gurus, entrepreneurs, coaches and practitioners can be interviewed by adopting different forms of standardized questions, both from a qualitative and a quantitative perspective. Investigations could implement martial classes on specific populations and monitor how they respond to the pedagogies of the practices in different ways (physically, mentally and socially).

By collecting visual and written documents, it is also possible to develop “discourse analysis” (Gee, 2010) on health and wellbeing prompted by different coaches and martial associations ― this method is indeed inspired by Michel Foucault’s theorisations and it is one of the main tools for conducting critical analysis from different theoretical perspectives, such as phenomenology, poststructuralism and pragmatism. Discourse analysis is a powerful tool for inquires that consider pedagogies over time; how discourses about health and wellbeing of established associations and coaches, for example, change in relation to the broader symbolic and political landscape. “Narrative analysis” (Sparkes, 2005) of personal involvement in MACS and health practices surrounding them is another prominent method, as seen in Stephens et al.’s study of British Capoeira practitioners’ injury narratives (this issue). Health and care can be approached from critical angles, detailing how certain techniques of the body impact on physical and psychological wellbeing, as well as how certain ideals of wellbeing are being boosted, interiorized, lived or contested by long-standing practitioners. This leads us to our final conclusions that bring together the different aspects of our theoretical framework and methodological approach for “martial arts, health and society.”

## Conclusion

In conclusion, investigations into martial arts and combat sports (MACS) have been increasing steadily over the last two decades, with specific projects focusing their attention on themes of gender, violence, pedagogy and embodiment. Some refer to this field as “martial arts studies” (Bowman, 2015), while others talk of “martial arts anthropology” (Cynarski, 2012), a revival of the 19th century science of armed and unarmed fighting (“hopology”) or even a “New Hopology” focusing on the armed traditions of combat (Ryan, 2020). Within this growing body of inquiry across interconnected areas of social science, there is a need for a critical understand of (un)healthy pedagogies; a complex topic which remains surprisingly underexplored currently. Our article has made a small contribution to the new line of inquiry into health, wellbeing and wellness that adds to the special issue on “martial arts, health and society.” It is not strictly within the realm of medical sociology, but it should be judged as adding to a new stream of research into the world’s fighting systems and cultures of combat (Brown et al., 2019) in terms of how one can envisage, study and critically assess the relationships between movement, practice and health.

Drawing on our previous reflections and the existing literature, we have provided a theoretical proposal with a twofold aim. First, we have conceptualized health and its pedagogies by moving beyond a reductionist biomedical paradigm to one focused on subjectivity as seen through the lens of care of the self. Second, we have set up a framework on *cultivation* in order to conduct future analysis on the pedagogies of health, so as to foster interdisciplinary inquiries in order to ask what and how martial arts could improve, or even act as an obstacle to, personal and collective health. We hence frame health and wellbeing pursued by MACS pedagogies as *forms of cultivation*, aiming at pursuing happiness and harmony with the selves ― practitioners’ bodies and minds ― the others, and the environment, as well as attempting to transform the living condition of several populations and the status quo to some extent, taking into account the social organisation of the practices and how they are experienced. Other valued qualities such as empowerment, self-control and social awareness could also be studied using the strands of our theoretical model. Investigations into values, dispositions and other aspects of humanity and society that are cultivated in MACS would add to knowledge on the cultivation of health.

We have added a few methodological suggestions for future analysis, considering the importance of this perspective given a few global trends, as the contemporary crisis of the Welfare-States (especially following the COVID-19 pandemic), the commodification of culture and the ageing process and the associated issue of isolation ― which affects Western countries in particular. We anticipate research across methodological paradigms, methodologies and traditions to provide a well-rounded subfield of martial arts and health that contributes to martial arts studies (or whatever the field might be termed in different languages and regions) and the social science/sociology of health, illness and medicine.

In summary, this article has contributed to the broad question “How might martial arts and combat sports be good/bad for health?” This complex question requires a multifaceted perspective that considers individuals, the relationships between them, wider sociopolitical dynamics and ecological perspectives beyond humanity. Martial communities can sustain individual and collective projects for improving wellbeing, as they can harbour individual health and progressive societal change. Specific research questions inspired by the theme of martial arts, health and society explored in this special issue and our particular article might include the following: Considering the fluid nature of martial activities between traditions and sports, how can goals of performance and health can be balanced in MACS experience? How do federations and coaches conceive health in the first place? To what extent could precise health pedagogics be part of coaching programmes and coach education? How do practitioners frame their wellbeing? Finally, bearing in mind the high dropout rate in martial arts clubs, to what extent do the (un)healthy pedagogies of the practices influence the practitioners’ involvement and withdrawal from MACS?

A lot of work has still to be done to move beyond this early collection of eclectic research projects seen in *Frontiers in Sociology*. Scholars across the world might wish to expand their research agenda to several different disciplines in order to compare and find general and specific traits of the different pedagogies, facing similar questions. Our proposal is an invitation to bridge different fields of inquiries so as to establish an open and critical dialogue across subjects with the ultimate goal of creating new research strategies and, hopefully, for policy making.

## Data Availability

The original contributions presented in the study are included in the article/Supplementary Material, further inquiries can be directed to the corresponding authors.

## Footnotes

1 Lorenzo lives in Milan, Italy, and has been practising boxing for four years. His latest publications focus on *boxe popolare* ― a boxing style widespread in Italy promoted by leftist grassroots groups. George is based in Wales, and he has a longstanding experience in several Eastern martial arts, Mexican Xilam and medieval fencing (historical European martial arts or HEMA), with numerous publications on several martial arts from throughout the globe.

2 The term ‘martial arts and combat sports’ (MACS) involve a myriad of styles and systems from all corners of the world: many remain unregulated; others are sporting activities; others are state-regulated exercises, or forms of military and civilian self-defence; some of them remain folkloric and vernacular arts.

3 To develop his (unfinished) project of tracking the ‘genealogy of subjectivity’ (Depew 2016, p. 24) ― revolving around the question of how subjects are established in different historical and institutional contexts ― Foucault looked back to the Ancient Greek civilisation, where morality became a topic of philosophical problematization and discussions (Foucault 2005; 2011).

4 To stress the philosophical dimension of the care of the self as a life-principle, Foucault analyses the ancient philosophies of Cynics, Stoics, and Epicureans. All those philosophical perspectives were indeed concerned with reaching a state of grace, happiness and wellbeing, criticizing the dominant ways of living and thinking in the polis at that time ― Foucault examines in particular the biographies and the writings of a limited amount of philosophers who lived in Ancient Greece and Rome (Foucault, 2005; Depew, 2016). In addition, according to Foucault’s thesis, all those philosophies constitute the basis for the care of the self as a general (critical) attitude throughout Western civilisation (see in particular: Foucault, 2011). A similar thesis evokes Dunning and Waddington’s (2003) differentiation between the stoic culture and the epicurean culture, calling for a more balanced experience in terms of pursuing health and wellbeing.

5 Several of Foucault’s analyses (Foucault, 2000; Foucault, 2005; Foucault, 2011) are devoted to the gymnastic exercises and the embodied activities prompted by philosophical schools. In his examinations, the philosophers thus appear to be as ancient gurus who share several features with the modern charismatic figures of the martial artist founders (cf. Brown, 2020) Accordingly, Pierre Hadot (1995) sustains that the main objective of the ancient Greek schools of philosophy did not consist in elaborating, or even the teaching, theories. On the contrary, they were more concerned about the transformation of individuals.

6 An example can be found in the scheme of this program https://www.guidestar.org/profile/26-2793142.

7 See https://www.bbc.co.uk/programmes/p08zkvjy.

## References

[B1] Abramson C. M. Modzelewski D. (2011). Caged morality: moral worlds, subculture and stratification among middle-class cage-fighters. Qual. Sociol. 34, 143–175. 10.1007/s11133-010-9175-8

[B2] Allen Collinson J. Owton H. (2015). Intense embodiment: senses of heat in women’s running and boxing. Body Soc. 21, 245–268. 10.1177/1357034X14538849

[B3] Allen Collinson J. Vaittinen A. Jennings G. Owton H. (2016). Exploring lived heat, "temperature work" and embodiment: novel auto/ethnographic insights from physical culture. J. Contemp. Ethnogr*.* (Online Early). 10.1177/0891241616680721

[B4] Alston R. (2017). Foucault’s empire of the free. Foucault Stud. 94–112. 10.22439/fs.v0i0.5246

[B5] Alter J. (1992). The wrestler's body: identity and ideology in North India. Berkeley, CA: University of California Press.

[B6] Bates C. (2019). Vital bodies: living with illness. Bristol, UK: The Polity Press.

[B7] Berni S. (1995). Foucault e le tecnologie del sé, Filosofia e Discussione Pubblica 8, 728–734.

[B9] Bourdieu P. (1985). The social space and the genesis of groups. Theor. Soc *.* 14, 723–744. 10.1177/053901885024002001

[B10] Bowman P. (2015). Martial arts studies: disrupting disciplinary boundaries. Lanham, MD: Rowman & Littlefield.

[B11] Bowman P. (2017). The definition of martial arts studies. Martial Arts Stud. 6–23. 10.18573/j.2017.10092

[B12] Bowman P. (2018). The martial arts studies reader *.* Lanham, MD: Rowman & Littlefield.

[B16] Brown D. H. K. (2020). Embodying charismatic affect(If): the example of Bruce Lee. Corpus Mundi 1, 14–52. 10.46539/cmj.v1i3.22

[B13] Brown D. H. K. Jennings G. (2013). “In search of martial habitus: identifying core dispositions in Wing Chun and Tajiquan.” in Fighting scholars: habitus and ethnographies of martial arts and combat sports, Editors Sánchez García R. Spencer D. C., London, UK: Anthem Press, 33–48.

[B14] Brown D. H. K. Jennings G. Pedrini L. (2019). Cultures of combat: body, culture identity. Etnogr. Ric. Qual. 12, 297–316. 10.3240/95526

[B15] Brown D. H. K. Jennings G. Sparkes A. C. (2014). Taijiquan the ‘Taiji World’ way: towards a cosmopolitan vision of ecology. Societies 4, 380–398. 10.3390/soc4030380

[B17] Bueger C. (2014). Pathways to practice: praxiography and international politics. Eur. Polit. Sci. Rev. 6, 383–406. 10.1017/S1755773913000167

[B22] Channon A. (2012). Western men and Eastern arts: the significance of Eastern martial arts disciplines in British men’s narratives of masculinity. Asia Pac. J. Sport Soc. Sci. 1, 111–127. 10.1080/21640599.2012.751170

[B23] Channon A. (2013). “‘Do you hit girls?’: some striking moments in the career of a male martial artist”. in Fighting scholars: habitus and ethnographies of martial arts and combat sports, Editors Sánchez García R. Spencer D. C., 95–110. London, UK: Anthem Press.

[B21] Channon A. (2014). Towards the ‘undoing’ of gender in mixed-sex martial arts and combat sports. Societies 4, 587–605. 10.3390/soc4040587

[B19] Channon A. Jennings G. (2014). Exploring embodiment through martial arts and combat sports: a review of empirical research. Sport Soc. 17, 773–789. 10.1080/17430437.2014.882906

[B18] Channon A. Matthews C. R. Hillier M. (2020). Medical care in unlicensed combat sports: a need for standardised regulatory frameworks. J. Sci. Med. Sport 23, 237–240. 10.1016/j.jsams.2019.10.014 31706826

[B20] Channon A. Phipps C. (2017). ‘Pink gloves Still give black eyes’: exploring ‘alternative’ femininity in women’s combat sports. Martial Arts Stud. 24–37. 10.18573/j.2017.10093

[B24] Cynarski W. J. (2012). Martial arts phenomenon: research and multidisciplinary interpretation *.* Rzeszów, Poland: Rzeszów University Press.

[B26] Delamont S. Stephens N. (2019). 'Capoeira is everything to me': British student commitment to the African-Brazilian martial art. Etnogr. Ric. Qual. 12, 365–384. 10.3240/95529

[B25] Delamont S. Stephens N. Campos C. (2017). Embodying Brazil: an ethnography of diasporic Capoeira. London, UK: Routledge.

[B27] Depew J. F. (2016). Foucault among the stoics: oikeiosis and counter-conduct. Foucault Stud. 22–51. 10.22439/fs.v0i0.5012

[B28] Dicks B. Flewitt R. Lancaster L. Pahl K. (2011). Multimodality and ethnography: working at the intersection. Qual. Res. 11(3), 227–237. 10.1177/1468794111400682

[B29] Dunning E. Waddington I. (2003). Sport as a drug and drugs in sport: some exploratory comments, Int. Rev. Sport Sociol. 38 (3), 351–368. 10.1177/10126902030383006

[B30] Durkheim É. Mauss M. (2010). Primitive classification. London, UK: Routledge.

[B31] Eichberg H. (1998). Body cultures: essays on sport, space and identity. Abingdon, UK: Taylor & Francis.

[B32] Elliot A. (2012). Reinvention. London, UK: Routledge.

[B36] Foucault M. (1983a). “The subject and power.” in Michel Foucault: beyond structuralism and hermeneutics *,* editors Dreyfus H. L. Rabinow P.. Chicago, IL: University of Chicago Press

[B33] Foucault M. (2000). Ethics: subjectivity and truth. Essential works of Foucault 1954-1984. Harmondsworth, UK: Penguin.

[B35] Foucault M. (2005). The Hermeneutics of the subject: Lectures at Collège de France, 1981-1982. New York, NY: Palgrave.

[B34] Foucault M. (2011). The courage of truth: the government of self and others II: lectures at the Collège de France 1983-1984. New York, NY: Palgrave.

[B37] Fuller C. Lloyd V. (2019). Martial arts and wellbeing. London, UK: Routledge.

[B38] Gee J. P. (2010). How to do discourse analysis: a toolkit. London, UK: Routledge.

[B39] Giddens A. (1991). Modernity and self-identity: self and society in the late modern age. Cambridge, MA: Polity.

[B40] Green K. Evers C. (2020). Intimacy on the mats and in the surf. Contexts. 19 (2), 10–15. 10.1177/1536504220920188

[B41] Hadot P. (1995). Philosophy as a way of life: spiritual exercises from socrates to Foucault. Oxford, UK: Blackwell.

[B42] Herrera-Valenzuela T. Valdés-Badilla P. Franchini E. (2020). High-intensity interval training recommendations for combat sport athletes during the COVID-19 pandemic. Rev. Artes Marciales Asiát. 15, 1–3. 10.18002/rama.v15i1.6230

[B43] Iftode C. (2013). Foucault’s idea of philosophy as ‘care of the self’: critical assessment and conflicting meta-philosophical views. Proced. - Soc. Beha. Sci. 71, 76–85. 10.1016/j.sbspro.2013.01.011

[B44] Jaquet D. Demésy A. Tzouriadis I. E. (2020). Historical European martial arts: an international overview. Jungju-si: international centre of martial arts for youth development and engagement (ICM). Available at: http://www.unescoicm.org/eng/library/bookreport.php?code=bookreport_eng&idx=6428&page=1&ptype=view>. (Accessed August 13, 2020)

[B46] Jennings G. (2010). Fighters, thinkers and shared cultivation: experiencing transformation through the long-term practise of traditionalist Chinese martial arts *.* PhD thesis, Exeter, UK: University of Exeter.

[B49] Jennings G. (2014). Transmitting health philosophies through the traditionalist Chinese martial arts in the UK. Societies 4, 712–736. 10.3390/soc4040712

[B48] Jennings G. (2019). “The light’ and ‘dark’ side of martial arts pedagogy: towards a study of (un)healthy practices.” in Exploring research in sports coaching and pedagogy: context and contingency, Editors Corsby C. L.T. Edwards C. N., (Newcastle, UK: Cambridge Scholars Publishing), 137–144.

[B47] Jennings G. (2020). Martial arts under the COVID-19 lockdown: pragmatics of creative pedagogy. Sociología Del. Deporte. 1 (2), 13–24. 10.46661/socioldeporte.5242

[B50] Jones S. (2004). The intelligent warrior: command personal power with martial arts strategies. London, UK: Thorsons.

[B51] Judkins B. Nielson J. (2015). The creation of wing Chun: a social history of the southern Chinese martial arts, New York, NY: State University of New York Press.

[B52] Kaibara E. (2008). Yojokun: life lessons from a Samurai. London, UK: Kodansha International.

[B53] Kasper D. V. S. (2009). Ecological habitus: toward a better understanding of socioecological relations, Organ. Environ. 22, 311–326. 10.1177/1086026609343098

[B54] Littig B. (2013). On high heels: a praxiography of doing Argentine tango. Eur. J. Women’s Stud. 20, 455–467. 10.1177/1350506813496397

[B55] Markula P. Pringle R. (2006). Foucault, sport and exercise: power, knowledge, and transforming the self. London, UK: Routledge.

[B56] Matthews C. R. Channon A. (editors) (2015). Global perspectives on women in combat sports: women warriors around the world. London, UK: Palgrave Macmillan.

[B57] Mauss M. (1973). Techniques of the body. Econ. Soc. 2, 70–88. 10.1080/03085147300000003

[B58] Mellor P. A. Shilling C. (1993). Modernity, self-identity and the sequestration of death. Sociol *.* 27, 411–431. 10.1177/0038038593027003005

[B59] Melucci A. (1996). Challenging codes: collective action in the information age. Cambridge, MA: Cambridge University Press.

[B60] Nardini D. (2016). *Gouren, La lotta bretone. Etnografia di una tradizione sportiva*. Cargeghe: Documenta.

[B61] Nardini D. Scandurra G. (*forthcoming*), Hand-to-hand sports and the struggle for belonging, Ethnography.

[B62] Orbach S. (2008). Bodies *.* London, UK: Profile Books.

[B95] Partikova V. Jennings G. (2018). The kung fu family: a metaphor of belonging across time and place. Rev. Artes Marciales Asiát. 15, 35–52. 10.18002/rama.v13i1.5462

[B64] Pedrini L. (2020). La Boxe Popolare. Etnografia di una Cultura Fisica e Politica. Aprilia, Italy: Novalogos.

[B63] Pedrini L. Brown D. H. K. Aimini G. (2019). Leading the left: sociability and the micropolitics of cultural reproduction in grassroots boxe popolare coaching. *Sport Educ. Soc.* (Online Early). 10.1080/13573322.2019.1672147

[B65] Randall J. Munro I. (2010). Foucault’s care of the self: a case from mental health work. Org. Stud. 31, 1485–1504. 10.1177/0170840610380809

[B66] Reckwitz A. (2002). Toward a theory of social practices: a development in culturalist theorizing. Eur. J. Soc. Theor. 5, 243–263. 10.1177/13684310222225432

[B68] Ryan M. J. (2016). Venezuelan stick fighting: the civilizing process in martial arts. Lanham, MD: Lexington books.

[B67] Ryan M. J. (2020). Armed combative traditions of Latin America and the Caribbean: a hopological overview. Rev. Artes Marciales Asiát. 15, 34–49. 10.18002/rama.v15i1.5948

[B69] Sánchez García R. Spencer D. C. (editors) (2013). Fighting scholars: habitus and ethnographies of martial arts and combat sports. London, UK: Anthem Press.

[B70] Sassatelli R. (2010). Fitness culture: gyms and the commercialisation of discipline and fun. New York, NY: Palgrave.

[B71] Silk M. Andrews D. Thorpe H. (2019). Routledge handbook of physical cultural studies. London, UK: Routledge.

[B72] Smith D. (2015). Foucault on ethics and subjectivity: ‘Care of the self’ and ‘aesthetics of existence.’ Foucault Stud. 135–150. 10.22439/fs.v0i19.4819

[B73] Sparkes A. C. (2005). “Narrative analysis: exploring the whats and hows of personal stories.” in Qualitative research in health care, Editor Holloway I., Maidenhead, UK: Open University Press, 191–209.

[B76] Spencer D. C. (2011). Ultimate fighting and embodiment: violence, gender and mixed martial arts. London, UK: Routledge.

[B75] Spencer D. C. (2012). Narratives of despair and loss: pain, injury and masculinity in the sport of mixed martial arts. Qual. Res. Sport Exerc. Health 4, 117–137. 10.1080/2159676x.2011.653499

[B77] Thompson G. (2010). Warrior: a path to self-sovereignty. Oxford, UK: Snowbooks.

[B78] Wacquant L. J. D. (2013). “Homines in extremis: what fighting scholars teach us about habitus.” in Fighting scholars: habitus and ethnographies of martial arts and combat sports, Editors Sánchez García R. Spencer D. C., (London, UK: Anthem), 193–200.

[B80] White R. (2014). Foucault on the care of the self as an ethical project and a spiritual goal. Hum. Stud *.* 37, 489–504. 10.1007/s10746-014-9331-3

[B82] Winchester D. Green K. (2019). Taking yourself into it: how and when accounts shape motivation for social action. Sociol. Theor. 37 (3), 257–281. 10.1177/0735275119869959

[B84] Yuasa Y. (1987). The body: towards an eastern mind-body theory. Albany, NY: SUNY Press.

[B83] Yuasa Y. (1993). The body, self-cultivation and ki energy. Albany, NY: SUNY Press.

[B85] Zarrilli P. B. (2000). When the body becomes all eyes: paradigms, discourses and practices of power in kalarippayattu, a south Indian martial art. Oxford, NY: Oxford University Press

